# 

**Published:** 1899-06

**Authors:** 


### BOOKS RECEIVED.

Atlas of Skin Diseases. By Prof. Dr. Franz Mracek, of Vienna. Edited by Henry W. Stelwagon, M. D., Clinical Professor of Dermatology, Jefferson Medical College, Philadelphia. Pp. 199, with sixty-three colored plates and thirty-nine full-page half-tone illustrations. Price, $3.50 net. Philadelphia: W. B. Saunders, Publisher, 925 Walnut Street. 1899.

A Review of Recent Legal Decisions Affecting Physicians, Dentists, Druggists and the Public Health. Together with a brief for the prosecution of unlicensed practitioners of medicine, dentistry or pharmacy, with a paper upon manslaughter, Christian science and the law and other matter. By W. A. Burrington of the New York Bar, Counsel of the Dental Society of the State of New York; Lecturer of Medical and Dental Jurisprudence in the New York College of Dentistry, and one of the collaborators in A System of Legal Medicine, by Allan McLane Hamilton and others, etc. Duodecimo, pp. 105. Price, 50 cents. New York: E. B. Treat & Co. 1899.

A Text-Book of Anatomy. By American authors. Edited by Frederic H. Gerrish, M. D., Professor of Anatomy in the Medical School of Maine at Bowdoin College. In one magnificent imperial 8vo volume of 915 pages, with 950 engravings in black and colors. Cloth, $6.50 net; flexible water-proof binding for the dissecting table, $7.00 net; full leather, $7.50 net. Philadelphia and New York: Lea Brothers & Co., Publishers. 1899.

Text-Book of Ophthalmology. By Dr. Ernest Fuchs, Professor of Ophthalmology in the University of Vienna. Second American edition, translated and revised from the seventh enlarged and improved German edition. By A. Duane, M. D., Assistant Surgeon Ophthalmic and Aural Institute, New

York. Octavo, pp. xvi.—860, with 277 illustrations. Cloth, $5.00; sheep, $6.00. Sold only by subscription. New York: D. Appleton & Co. 1899.

Transactions of the Rhode Island Medical Society. Volume V. Parts III., IV. and V. Providence, 1897-98.

A Treatise on Human Physiology. For the use of students and practitioners of medicine. By Henry C. Chapman, M. D., Professor of Institutes of Medicine and Medical Jurisprudence in the Jefferson Medical College of Philadelphia. New (second) edition, thoroughly revised. In one handsome 8vo volume of 921 pages, with 595 engravings. Cloth, $4.25 net; leather, $5.25 net. Philadelphia and New York: Lea Brothers & Co. 1899.

Materia Medica and Therapeutics. An Introduction to the Rational Treatment of Disease. For the use of students and practitioners of medicine. By J. Mitchell Bruce, M. D., F. R. C. P., etc., Physician and Lecturer on Medicine at Charing Cross Hospital, London. New (sixth) edition, revised and enlarged. In one 12mo volume of 618 pages. Cloth, $1 50 net. Philadelphia and New York: Lea Brothers & Co. 1899.

An Epitome of the History of Medicine. By Roswell Park, A. M., M. D., Professor of Surgery in the Medical Department of the University of Buffalo, etc. Based upon a course of lectures delivered in the University of Buffalo. Second edition. Illustrated with portraits and other engravings. Pp. xiv.—370. Extra cloth, $2.00 net. Philadelphia: The F. A. Davis Co., Publishers, 1914-16 Cherry Street. 1899.

The Medical Complications, Accidents and Sequelæ of Typhoid or Enteric Fever. By Hobart Amory Hare, M. D., B. Sc., Professor of Therapeutics in the Jefferson Medical College of Philadelphia, Physician to the Jefferson Medical College Hospital, Laureate of the Medical Society of London, of the Academie Royale de Médecine de Belgique, etc. With a special chapter on the Mental disturbances following typhoid fever. By F. X. Dercum, M D., Clinical Professor of Diseases of the Nervous System in the Jefferson Medical College. Octavo, 267 pages, twenty-one engravings and two full-page plates. Cloth, $2.40 net. Philadelphia and New York: Lea Brothers & Co. Publishers. 1899.

